# Experiences of siblings to children with autism spectrum disorder

**DOI:** 10.3389/fpsyt.2022.959117

**Published:** 2022-07-25

**Authors:** Naledi Mokoena, Anwynne Kern

**Affiliations:** Department of Psychology, School of Human and Community Development, University of the Witwatersrand, Johannesburg, South Africa

**Keywords:** autism spectrum disorder, South Africa, ASD, experiences, family stress, coping, development

## Abstract

Autism spectrum disorder (ASD) is prevalent globally resulting in increased awareness and understanding of the disorder internationally and to a lesser extent in Southern African countries. This understanding has predominantly been in relation to the impact of the disorder on the neurodivergent child and family relations. Internationally, limited research has explored the impact on neurotypical siblings who have been raised alongside children diagnosed with ASD, with a dearth of such studies emerging from the South African context. The importance of neurotypical siblings’ developmental experiences emerging from their immediate environment is significant within the traditionally collectivist nation of South Africa. For this reason, this study aimed to explore South African neurotypical siblings’ experiences of living with a brother or sister diagnosed with ASD; through a qualitative study adopting an interpretative phenomenological design. Semi-structured interviews, aimed at generating demographic data and exploring the experiences of being raised alongside a sibling diagnosed with ASD, were conducted with eight university students. The data generated were analyzed employing the five stages of interpretive phenomenological analysis approach. Themes of emotional burden, perceived family dynamics, acceptance, and identity development emerged through data analysis. The participants reported experiences of pre-mature development, unfair treatment, and feelings of being overburdened, along with reported efforts toward acceptance of their sibling’s diagnosis, and support from community members. Consequently, an understanding that their neurodivergent sibling played a key role in the development of their identities and career trajectories emerged. The impact of being raised alongside a sibling diagnosed with ASD highlights the need for additional support for neurotypical individuals, supported by programs to enhance awareness about ASD in the general South African community. These interventions would be aimed at mitigating the impact of heightened childhood stress, stigmatization, and marginalization.

## Introduction

Autism spectrum disorder (ASD) is prevalent globally with incidence numbers reported at 1 in every 160 children in the world (1) and 1 in every 59 children in the United States (2). While statistics have not been finalized in South Africa, it is estimated that similar prevalence rates exist as compared to the United States (3). As a result, there is increased research attention on understanding the effects of ASD in children diagnosed with the disorder, along with those living with them (4).

Research has shown that within Africa, there are low levels of knowledge and awareness with regard to ASD and its effects, implying that intervention and support systems are limited (5, 6). The inadequate dispersion of knowledge with regard to ASD in psychology training is reported by Ruparelia et al. (6). Furthermore, these researchers note that where there is training, minimal treatment and supportive services are available. Similarly, South Africa too placed minimal emphasis on the disorder within the health sector, despite a shift toward offering support services to parents soon after the initial diagnosis (7). Research conducted by Mchunu (8) and Chambers et al. (9) indicated that residents of two South African cities had limited awareness of ASD, its effects on diagnosed children, and those living with them, implying a limited understanding of the disorder and its impact among the South African population. In addition, individuals diagnosed with the disorder were stigmatized.

Internationally, research has explored the effects of living with an ASD-diagnosed child over the past two decades. Researchers have revealed teachers’ perspectives (10); and assessed the dynamics of families living with children with ASD, with a strong emphasis being placed on the parents’ experiences (11, 12). In the African context, the previous studies assessed the experiences of families of children with ASD (4). While noting the importance of these previously focused research samples, limited research explores the experiences of neurotypical siblings within families.

Psychological research stresses the nexus between children’s environments and their development. In his theory, Bronfenbrenner argues that the power of the home or family as part of the microsystem lies in the types and quality of interactions that occur between children and other members within their microsystem over time (13, 14). This indicates that the bidirectional relationship between ASD children and their neurotypical siblings are crucial for the siblings’ developmental processes within their contexts. Siblings living within children’s microsystems contribute toward their foundational social interactions, knowledge, life interests, and values (15). Consequently, for this research, the neurotypical sibling’s experiences are primal concerns as a trait of their development may be a result of having a sibling on the autism spectrum.

A distinct nature of the African climate is its collectivism as opposed to Western nations that are more individualistic (16). In South Africa, the immediate and indirect social surroundings influence how children introject their daily experiences and attach meanings to them (17–19). Collectivism translates to children’s identities being more dependent on their immediate environments as opposed to Western nations where individuals are inclined toward independence (20). These ideas suggest that in South Africa, the ASD-diagnosed children and their neurotypical siblings will largely influence each other’s developmental experiences. This highlights that experiences of siblings living with children diagnosed with ASD in the South African context require more research attention as familial and other social experiences could have a significant influence on them and their development. This study therefore aims to gain insight into the experiences of South African late adolescents and emerging adults with ASD diagnosed siblings.

## Brief overview of autism spectrum disorder

Autism spectrum disorder, also known as autism, describes a neurodevelopmental disorder with symptoms mainly manifesting from the age of two. Diagnosis is, however, more prevalent from middle childhood with an increase in academic and/or social demands for children (21). According to the *Diagnostic and Statistical Manual of Disorders—Fifth Edition* (DSM-5), the children diagnosed with ASD demonstrate atypical symptoms such as an inadequate understanding of social cues and deficits in communication (22). They also display repetitive behaviors, activities, and interests; and may exhibit resistance to change (22, 23). Some persons display an increased ability in rote memory, may experience eating and/or sleeping difficulties, display psychopathology, and related health conditions (21, 24).

Autism spectrum disorder presentation in individuals lies on a continuum, ranging from Level 1 to Level 3, based on the levels of support required by the individual across the domains of cognition, language and behavior (22, 25). Individuals placed on Level 3 require substantial support and present with severe deficits in social communication, leading to limited initiation and interactions with others. They also display monotonous behaviors that disrupt standard operations in all facets of life. Level 2 is characterized by individuals who display simple means of verbal and non-verbal communication thus requiring substantial support. These individuals struggle to cope with change and exhibit repetitive behaviors apparent enough to a casual observer. Individuals placed at Level 1 require some support and they struggle to make friends. Even though they are capable of having logical conversations, those diagnosed under this level have tedious manners and self-organization challenges that affect day-today functioning.

While acknowledging the impact that ASD has on the diagnosed individual, research also demonstrates the impact that the disorder has on the individuals in the microsystem, with this study specifically focusing on the neurotypical sibling.

## Autism spectrum disorder and the neurotypical sibling

While there is a dearth of literature studying the experiences of neurotypical siblings of children diagnosed with ASD, the research that has been conducted are predominantly located within the Euro–American context (11, 26–32). In these studies, findings stated that neurotypical children reported positive as well as adverse influences on social competence and self-concept due to having a sibling on the spectrum. Having said that, externalized and internalized behavior in neurotypical siblings predominantly increased. Families with ASD children have reported (31, 32) that sibling relationships are often strained as neurotypical children that have reported being overburdened by consequential familial responsibilities (26, 32). Similarly, in South Africa, responsibility of caring for younger siblings was found to be given to older siblings and less dependent on adult care (9). These responsibilities are understood as emerging from parents’ feelings of inadequacy in dealing with their children with ASD singlehandedly in the Kapp and Brown (4) study conducted in South Africa.

Feelings of neglect from family members and financial struggles, resulting from the family resources being used on the therapy for the child diagnosed with ASD, was reported by the neurotypical siblings in a study conducted by Hastings (33) in the United Kingdom. Feelings of embarrassment and shame were also narrated by Petalas (31), negatively affecting friendships, including the parental and sibling relationship (26, 34). With regard to the parental relationship, neurotypical siblings placed less reliance on parental guidance reporting higher levels of independence, as a strategy to maintain the family cohesion while achieving their personal life goals. Thus, as indicated by Chan and Goh (35), neurotypical siblings are influenced by the microsystems in which they develop, along with their personality characteristics. For example, neurotypical siblings displayed heightened levels of compliance or resistance to daily family dynamics. Noteworthy, despite being aware of their siblings’ limitations early in life, a mature understanding of how their sibling’s ASD affected their own and their siblings’ lives (27, 29, 31) emerged as the participants grew older.

Neurotypical siblings to children with ASD also reported having positive experiences related to their siblings’ ASD. Closer relationships to their parents were reported by neurotypical children in the Orsmond and Seltzer (29) study conducted in the United States. In Greece, neurotypical adolescent siblings of children with ASD reported increased responsibility, better school adjustment, and increased self-concept as a result of their sibling’s diagnosis (32). Furthermore, some siblings to children with ASD did not experience adjustment challenges as compared to the siblings of typically developing children as discussed in the study by Ferraioli and Harris (34). Noting this, Hodapp and Urbano (28) highlighted that some nations may place different emphasis on the sibling role within families, highlighting the impact of different cultural experiences and its impact.

Having discussed the elements related to ASD, the attention will now be turned to the theories underpinning this study.

## Theoretical overview

As a means of analyzing the varied experiences and coping mechanisms of neurotypical siblings to children with ASD, the research employed both bio–ecological theory and Lazarus’ Cognitive Mediational Model of Stress and Coping.

### Bio–ecological theory

**FIGURE 1:**
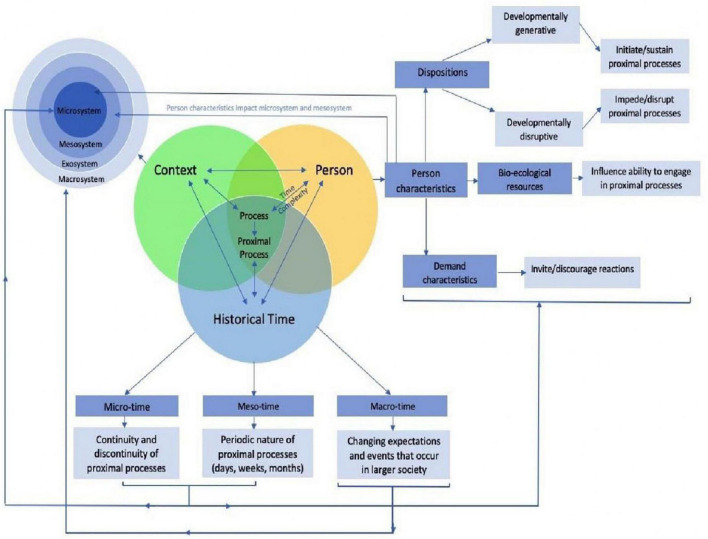
Bio–ecological model (38).

Bronfenbrenner’s bio–ecological theory, having emerged in 2007 (36), is aimed at understanding the developmental and systemic factors affecting the development (37), through four dimensions; process, person, context, and time. As reflected in Figure 1 (38), these dimensions influence development simultaneously, with the process dimension central to the theory.

The process dimension located centrally captures the duality of the interactions between the person and context across time (13). The interactions that occur between the individual and people, objects or symbols are referred to as proximal processes. For the purposes of this study, the neurotypical sibling is placed at the center of the model, with their proximal processes, and factors affecting those processes, under study. However, the individuals are not powerless within their environment, instead influencing their interactions with others through their personality characteristics.

Through their influence on the direction and power of proximal processes, the person dimension of the theory influences future development (39), and consists of dispositions, bio–ecological resources, and demand characteristics (13, 39). With regard to the neurotypical individual, these personality characteristics will, for example, influence the way they respond to peers teasing them because they have a sibling with ASD (ignore or retaliate) or the type of support that they receive from their microsystem (teachers or faith members lending a listening ear). It is important to note that the personality characteristics of the individuals in the microsystem and mesosystem also influences proximal processes. For example, the beliefs held by peers regarding the etiology of disability, while influenced by the macrosystem, informs the interaction with the neurotypical sibling in the microsystem, possibly resulting in marginalization and stigmatization of not only the sibling with ASD but the neurotypical sibling.

The context dimension of the bio–ecological theory is widely known and refers to the microsystem, mesosystem, exosystem, and macrosystem. The microsystem refers to the immediate environments and settings that the neurotypical individual has contact with, e.g., parents, family, neuroatypical sibling, teachers, and peers. The mesosystem refers to the interactions between individual settings found in the microsystems, such as the relationship between the teacher and parents. The exosystem refers to those environments which affect the neurotypical individual either directly or indirectly, but which do not interact directly with the individual. An example may be the neuroatypical sibling’s therapist who decides on increasing the frequency of the therapy. While the neurotypical individual has no contact with the therapist, the decision in the exosystem implies that the parents are away more often, and funds are further depleted. Finally, the macrosystem refers to the beliefs, ideologies, customs, traditions, and policies found in the cultural world surrounding the individual (39–41). Specifically, health and education policies are located within this system, as are beliefs regarding disability and mental illness. Thus, the macrosystem influences the development of values and beliefs.

The bio–ecological theory is subsequently used in this study to understand the nature and factors affecting the interactions (proximal processes) between the neurotypical individual within their various contexts. However, missing from this theory is the way the neurotypical siblings made sense of their experiences. Consequently, the Cognitive Mediational Model was also used to understand the findings in this study.

### Stress and coping: The cognitive mediational model

**FIGURE 2:**
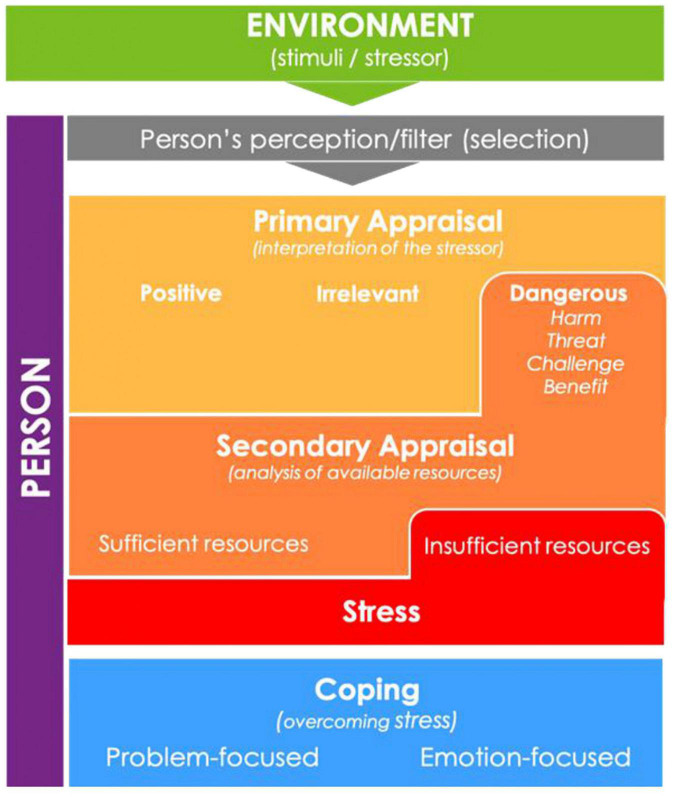
Illustration of Lazarus Cognitive Mediational Model (42).

The use of the Cognitive Mediational Model does not assume that families with ASD diagnosed individuals are universally stressed. Rather the theory assists in explaining the research participants’ childhood experiences and coping mechanisms. Further is a discussion and illustration (Figure 2) of the Cognitive Mediational Model and its features.

Lazarus (43) highlighted that people are active agents in both admirable and unadmirable life experiences. He explains that through their cognitive, emotional, and behavioral traits, individuals make choices about the impact that experiences, particularly stressors, have on their lives. This idea is similar to Bronfenbrenner’s notion of personality characteristics. Lazarus (43) defined stress as a transaction between individuals and their environments that is perceived as personally significant. Experiences of stress are thus not universal and cannot be generalized. While research has demonstrated that families with a child diagnosed with ASD undergo stress (44), the nature and experience of that stress needs to be understood from the individual’s standpoint, highlighting the importance of exploring individuals’ experiences. In this regard, understanding perceived protective and risk factors, and their role in the development of coping strategies and resources within the environment is important.

Fundamental to the Cognitive Mediational Model is the idea that when individuals go through stressful experiences, they evaluate these occurrences according to the influence they might have on their well-being (45). This unconscious evaluation process is referred to as appraisal. In appraisal, individuals negotiate between the demands, constraints, and resources that are associated with the concerned stress. Individuals weigh these against their personal goals and beliefs and through this process, they mediate their response to stress according to their subjective perception (46). The unconscious interpretations result in people’s subjective cognitive and emotional reactions to stress related situations (47). It can be assumed that neurotypical individuals go through the process of appraisal, in relation to their diagnosed sibling, fairly frequently, with their subjective experience informing their response. These daily stressors related to their siblings’ diagnosis, as opposed to major life events, are understood to be predictors of their psychological states (45).

As individuals appraise their life events—whether they be favorable, stressful, or traumatic—they also navigate coping. Lazarus and Folkman (48), (p.141) perceived coping to be the “constantly changing cognitive and behavioral efforts to manage specific external and/or internal demands that are appraised as taxing or exceeding the resources of the person.” Coping is not a definite way of being but rather a process that needs to be circumstantially navigated (46). Additionally, different strategies of coping may be applicable in varying situations (49).

According to the Cognitive Mediational Model, coping styles can be categorized into either problem-focused or emotion-focused coping. In problem-focused coping, individuals purposefully intend to modify the situation between themselves and their environments. They take physical actions that can potentially improve the environmental stressor. In emotion-focused coping, individuals change from negative perception of their experience and perceive it as agreeable within parameters of their control (50). As noted by Lazarus (46), some coping strategies may be more effective than others. For instance, persons with higher internal loci of control can successfully use positive attitudes as frequent means of coping. Hence, their personal characteristics afford them the opportunity to engage in emotion-focused coping in dealing with life stressors more often. However, people who rely on environmental changes for coping may be less amenable to coping as their strategies will be reliant on external factors and this gives them a lower perception of control over their stressors (49). Lazarus’ model of stress and coping assists in ascertaining the meaning of participants in this study made of childhood experiences, both those perceived as positive and challenging.

Nothing that there are low levels of knowledge in Africa with regard to ASD, this research therefore looks at the influences of having a sibling with the disorder. In this way, the daily interactions related to ASD on neurotypical siblings can indicate how the country’s collectivist values affect development.

## Materials and methods

### Study design

The study adopted an interpretive phenomenological analysis research design, not only to understand the experiences neurotypical siblings had in relation to their sibling diagnosed with ASD but also to understand how they made meaning of their experiences (51). Due to the dearth of research on the topic in the South African context, an in-depth understanding of the phenomenon was sought, the results of which may be used to inform future research.

### Sample and sampling

Due to the aim of the study, a purposive sampling technique was used to solicit participants who met the inclusion criteria. To be considered for inclusion in the study, the participants must have a biological sibling diagnosed with ASD and have lived/grown up in the same home with their sibling. Confirmation that the participants met the inclusion criteria was sought as part of the data generation process.

Using data saturation as a means of determining sample size (52), the sample size consisted of eight university students from one university’s Faculty of Humanities. Given that we aimed to study a few individuals in extensive detail (53), along with data saturation, eight participants were deemed sufficient. The research sample consisted of eight females (100%). The mean age of the participants was 20.8 years with an age range of 19–25 years. Given the age range of the participants, their developmental stage is characterized by identity formation, having moved beyond the tumultuous season of mid-adolescence (54). The participants were thus thought to have the cognitive capacity to reflect on and share how their childhood experiences shaped their identity development, identity closure, and life course (55).

Majority of the sample identified as Black (*n* = 5, 62.5%), while 12.5% (*n* = 1) of the participants identified as colored, White, and Indian, respectively. This was indicative of the statistics of the Johannesburg population, with its residents comprising 72% Blacks (56). Similar statistics emerge in relation to household income, with 62.5% (*n* = 5) of the participants indicating that they grew up in homes with dual incomes, with the remaining 37.5% (*n* = 3) participants noting that their homes had only one income. The diverse socioeconomic backgrounds of the participants would influence the availability of resources, and exposure to and understanding of ASD.

The participants’ siblings with the ASD diagnosis were predominantly male (*n* = 7) and one female. As discussed in the previous sections, ASD symptoms present on a spectrum with different individuals experiencing different severity of symptoms. Based on information provided by the participants and according to the DSM-5 criteria, 37.5% (*n* = 3) of participants had siblings who were understood to have Level 1 ASD, 25% (*n* = 2) had Level 2 ASD, and 37.5% (*n* = 3) were understood to have Level 3 ASD. The participants who had siblings with Level 3 ASD were involved in the physiological care of their sibling, such as assisting with washing and feeding. The level of ASD experienced by the sibling is important to note as it has implications for the responsibilities the neurotypical sibling may have to fulfill. Siblings were diagnosed with ASD at different ages with participants learning about the diagnosis 7–22 years preceding the time of data collection. This indicates the amount of time they had been exposed to said responsibilities. The age of siblings with ASD ranged from 10 to 48 years, with a mean age of 22.7 years at the time of data generation. Most of the participants were older than their sibling diagnosed with ASD (*n* = 5, 62.5%).

### Measurement tool

The data was generated using semi-structured interview schedule (Appendix) in interviews with an average time of 1 h. In this way, the research could guide the topic of conversation, while still allowing the participants the opportunity to respond widely (57). The semi-structured interview schedule was developed based on the study aims as well as questions used in the Hodapp and Urbano (28) study exploring American siblings’ experiences to children with ASD. Questions from Glasberg’s study (27) were also used. Where Hodapp and Urbano (28) inquired about the activities that the sibling with ASD was able to perform, the researcher inquired about the activities and household chores that all children in the house completed. The data regarding how the participants perceived their lives would have been different had their sibling not been diagnosed with ASD was drawn from “The Implications of Autism Protocol” used in the Glasberg study (27), p.154).

The interviews first obtained the demographic information from the participants. These were used to understand the participants’ context. The demographic questions also confirmed that the participants had a sibling with ASD and that they fit all the inclusion criteria. The demographic differences were obtained to determine if varying backgrounds would offer differences among participants. Thereafter, interviews explored the relationship dynamic between participants and their siblings with ASD, how the disorder influenced their familial and social lives, and if/how participants imagined their lives would be without the influence of ASD. When participants shared additional information different to main interview questions, further probing questions were offered where relevant.

### Procedure

University ethics approval was granted from the ethics committee (MEDP/17/005 IH), followed by permission from the Faculty of Humanities’ graduate studies committee. An email invitation to participate in the study was then sent to the student emails of all students registered in the Faculty of Humanities, requesting that they make contact with the researchers should they have a sibling diagnosed with ASD and be willing to participate in the study. The participant information sheets were subsequently emailed to the students indicating the research purpose and process. Face-to-face interviews were then arranged with the participants at a time and place convenient for them. Prior to conducting the interviews, signed written consent was obtained for participation in the study as well as for audio-recording. It was anticipated that the voluntary nature of the study would allow the participants to share their experiences candidly and openly. Given the nature of the interviews and the experiences discussed, debriefing occurred at the end of the interviews. The audio recordings were transcribed verbatim subsequent to the interviews.

Once the interviews were conducted, notes were made relating to additional observational information noted during the interview. Audio-recordings were transcribed verbatim, maintaining the original language used in interviews, that being English, and isiZulu for one participant. The transcriptions were stored on a password-protected computer for the data analysis, as discussed in the following section; and transcriptions were only accessible to the primary researcher. Relevant demographic information was also collated to establish the research context and influence on data findings, hence ensuring transferability of the research process. In maintaining credibility of the data, all quotes used in the discussion chapter are verbatim as transcribed from interviews. Furthermore, in this article, the context of the participants and thick descriptions are included to show how the process of the research can be transferred to other contexts. Thick data relates to aspects such as participant feelings, that gives a greater understanding of participants meaning making (58). Efforts toward dependability were made through collaboration with a research colleague, thus ensuring validation of the research process. Additionally, the researcher engaged in reflective practices during data collection, analysis, and write-up. Those included awareness of their identity, beliefs, and biases and how these may have influenced their epistemology (52). One such reflection relates to the participants experiences with therapists, given the therapeutic background of the principal investigator. Such awareness was highlighted when participants confirmed at the onset of interviews the intentions of the research, as a means of mitigating against stigmatization of their siblings with ASD.

### Data analysis

It could be argued that the interpretation of data is where Interpretive Phenomenological Analysis’s (IPA) relevance lies. Consequently, while an exploration and description of participants’ experiences is required, an interpretation of the findings and communication of what meaning was placed on their lived experiences is necessary (59). In line with this, the participants’ experiences could not be taken as a generalization, but as providing clarity on the potential experiences of neurotypical siblings whose sibling is diagnosed with ASD in the South African context; this despite the themes that were generated from the data.

The data generated was read, understood, and interpreted using the IPA approach. By using this approach, the researcher aimed to “enter the life world” (60), p.286) of the participants to get as close as possible and gain insight into what people make out of their lived experiences (61). The five stages of the IPA framework were implemented in the data analysis (60).

First, the researcher read all written text from interviews, i.e., transcripts and notes made after interviews. Thereafter, information that was of interest to the study, was highlighted, also known as a process of coding. Second, the codes were then cataloged into themes or patterns—both overarching and specific to individuals’ experiences. In this process, 18 detailed themes were identified, and categorized under 9 overarching themes. Additional significant information was also highlighted. Third, following the identified themes, a structure of presenting the findings was developed guided by the research questions. This means that presented themes responded to questions and aims of the research. Fourth, themes identified were summarized into 12 global themes, of which 6 were dominant. Of these, five were chosen to be included in this article, due to their relevance. Lastly, identified data and insights were integrated into the results and discussion chapter of the research report. While analyzing the data using the IPA framework, the researcher consistently interpreted the human experience obtained from participants, keeping true to the descriptive and interpretivist nature of IPA.

Texts from the participants’ responses provided insight into their lives to depict them to the reader. It should be noted that in using IPA, the researcher acknowledged that the spoken words of the interviewees were also a result of their cognitive experiences in relation to their neurodivergent sibling (61). The participants’ perceptions can be accepted as a means of further understanding the possible realities of South African siblings to children with ASD.

## Results and discussion

In keeping with the research aim, interviews conducted were analyzed to assess if neurotypical children had experiences that they felt were caused by living with a sibling diagnosed with ASD. Analysis also looked at how participants’ experiences affected their development, their familial dynamic, and their social selves. Five superordinate themes emerged, namely, Emotional Burden, Perceived Family Dynamics, Social Consequences, Acceptance, and Identity Development. These, along with the subordinate themes are presented in Table 1.

**TABLE 1:** Superordinate and subordinate themes.

Superordinate themes	Subordinate themes
Emotional burden	Emotional struggles Conflicted feelings
Perceived family dynamics	Unfair treatment between siblings Level of support Parenting their siblings
Social consequences	
Acceptance	Normalization The role of faith
Identity development	ADS as core to identity development Influence on chosen life path

### Emotional burden

Not unexpected, the participants reflected on the emotional aspect of growing up alongside a sibling diagnosed with ASD. Reports of being emotionally overburdened were reported by the neurotypical siblings. This resulted in a cycle of emotional struggle and conflicted feelings related to their siblings.

#### Emotional struggles

Similar to the previous studies (11, 35), the participants in this study note the emotional impact of having a sibling diagnosed with ASD. The previous studies (26, 29, 32) highlighted the feelings of stress associated with having a sibling on the spectrum; while the participants (*n* = 5, 62.5%) in this study reported feelings of frustration, sadness, and exhaustion. Most of the participants (*n* = 7, 87.5%) indicated that having a sibling on the spectrum caused them emotional distress during their childhood and recently in their late adolescent years.

“It was frustrating. It was irritating having someone like him in the house” (Participant 6).

“At some point you actually feel like you know what I hate this person… for having to become the center of attention…for not being my older brother. I hate the fact that I need to sit at home and feed you whereas I could be playing with my friends. I hate the fact that my mom can’t afford to for instance take me to a better school, whereas you could have been grown and working and paying for my education” (Participant 7).

“It’s just like pain that you step on…but it goes with you…. Let’s say it’s like gum on your shoe and I’m just walking. But like inside it hurts” (Participant 5).

The excerpts highlight the idea that the emotional challenges experienced by the neurotypical sibling was carried with them through the years and was not a singular event. This is linked to the responsibilities that the participants were given, along with the very idea of having a sibling with ASD and witnessing them going through daily life challenges related to the disorder. In this regard, the participants noted:

“I feel sorry because she will never get to experience life the way we are. She will never go to high school. She will never go to varsity. I don’t think she’ll get married like. (Sigh) she’ll never experience what we’re experiencing. She will never have a boyfriend. She will never have a first kiss” (Participant 8).

“Like this person is dealing with so much you know my brother, he’s going through the most. Like he did not have a normal life more than any of us” (Participant 4).

With regard to responsibilities, the participants noted that the additional responsibility that came with having a sibling on the spectrum resulted in feelings of being restricted from fully experiencing their childhood. For the older sibling, they were tasked with the care of someone else for most of their lives. These perceived limits to freedom resulted in feelings of stress (45), and powerlessness, despite the attempt to apply emotion or problem-focused coping. The participants noted, “You’re not really free” (Participant 8), “You can’t like be spontaneous with autism” (Participant 4), and “It wasn’t fun, and looking back, there was a lot taken from me as a child” (Participant 7). A similar finding was reported by Bitsika, Sharpley and Mailli (26), where a group of Australian children felt inhibited from fully living their life due to having to care for their siblings diagnosed with ASD. This indicates that South African children, similar to those in the Australian context, are required to care for their ASD sibling.

#### Conflicted feelings

The participants in the study reported experiencing the conflicting emotions of frustration and anger, and empathy toward their neurodivergent sibling. To resolve this emotional conflict, the participants employed emotional-focused coping, as explained in the Cognitive Mediation Model (50). Here, the participants acknowledged the stress they were under, while simultaneously acknowledging that their sibling with ASD experienced their own challenging life experiences. Participants 4 and 5 reported that as children, they felt compassion for their siblings, acknowledging that their brother or sister did not bring ASD onto themselves. Their emotion-focused coping strategy involved not casting blame onto their sibling because they remembered that, “…these kids they didn’t do anything” (Participant 4). Participants were thus able to separate the individual from the disorder, identifying that their feelings of anger and frustration is a result of the disorder itself (50). This is demonstrated in the following extract: “Me being pissed at the fact that it had to be him. Pissed at the fact that he would, he has to struggle so much. Pissed at the fact that he… doesn’t enjoy life like other people. I’m not really pleased with the fact that… he has autism. So… it is quite upsetting at times, it leaves one quite angry” (Participant 7).

While acknowledging the distress that the disorder caused them personally, the participants also noted that they did not share these feelings with their parents, instead choosing to deal with the feelings by themselves, “You would deal with everything else as long you don’t sound like… you’re blaming it on him” (Participant 6). This included expressing feelings of irritation and frustration that emerged because of failed communication with their sibling with ASD. The reasons behind not sharing their feelings of frustration and irritation with their parents stemmed from ideas of wanting to protect their parents, reducing their feelings of annoyance, and preventing or reducing the conflict within the home.

“I also don’t wanna show her (my mother) my emotions because I feel like I’ll upset her cause I mean she is pretty strong about it but…then she’ll think, oh okay so if you still this emotional then it means I’m not doing something right. So I don’t want her to feel like that” (Participant 2).

These results are supported by Chan and Goh (35) and Petalas et al. (30) who found that neurotypical children experienced ambivalent feelings toward their sibling diagnosed with ASD. The participants reported feeling the need to ease their parents’ stress (30), while those in the Chan and Goh (35) study negotiated emotions to maintain cohesive relationships with their parents. The conflicted feelings are argued to be a consequence of children regulating and buffering stressful emotions that they feel they should not have toward being a neurotypical sibling to a sister or brother on the spectrum (62). Similar to participants in the Singaporean study, South African children internalize their frustration to prioritize familial cohesion and relieve their parents’ pain. These being traits that may emerge as a result of growing up in a predominantly collectivist nation. In this way the personality characteristics of the individual are influenced by the proximal processes with their sibling.

### Perceived family dynamics

The impact of having a child with ASD on the family dynamics was noted by five participants (62.5%). In this study, the participants reported both positive and negative experiences. These experiences were categorized into ideas of being treated unfairly, having to develop pre-maturely; contrasted by feeling sufficiently supported by their parents.

#### Unfair treatment between siblings

The participants who had siblings with lower support needs reported that their neurodivergent siblings were perceived as less able to carry out daily activities than the participants believed. The APA (22) states that children with severe ASD (Level 1) require significant care and cannot carry out most selfcare functions, while the individuals with lower support needs, referred to by some participants as “high functioning,” have more cognitive and physical capacity to participate in daily household activities (22). In this regard, the personality characteristics in relation to the severity of autism symptoms influenced how the participants experienced their siblings. The differential treatment of the neurodivergent sibling was questioned in terms of the division of household responsibilities along with the general treatment received from parents or guardians.

“He [my sibling with ASD] knows he can manipulate her [my mother] … It’s not that he can’t do it, he’s lazy to do things and he manipulates her, so she feels bad for giving him more responsibility even though I’ve told her you know he’s manipulating you” (Participant 2).

“He got a lot of stuff that I didn’t get when he got it at his age…I was like just cause he has autism now he must get things that I didn’t get when I was his age—totally unfair, he should wait too” (Participant 5).

This finding correlates with reports from Stampoltzis et al. (32) where typically developing children perceived their siblings as having received unfair favorable treatment due to their disorder, with reference to the uneven allocation of household responsibilities. The finding that responsibilities are delegated unevenly appears to be consistent across contexts, indicating that children across both Western contexts and in South Africa, are likely to note the discrepancy in treatment between themselves and their sibling with ASD.

#### Level of support

The level of support received from parents was an additional sub-theme that emerged. While perceiving that they received differential treatment as compared to the neurodivergent sibling, the participants (*n* = 5, 62.5%) in this study noted that they felt supported by their parents and guardians, with regard to personal matters and challenging experiences. Instead, when reflecting on how their parents dealt with their challenges, the participants felt that they were comforted when needed, received the necessary support, and received guidance from their parents, indicating that the parents’ personal characteristics were able to support the proximal processes between the neurotypical sibling and themselves. The participants added that they were equivalently and adequately loved compared to their siblings diagnosed with ASD.

“When I think back as to how it was handled and how it went through, I really was well supported and it [support] was always there for me and I was made to be okay” (Participant 1).

“My mom was very supportive. I mean she loved us as we were all her children. So, I wouldn’t say she loved one more than another” (Participant 6).

Interestingly, 50% (*n* = 4) of participants reflected that while they did not feel supported growing up, reflecting back as young adults, this perception has changed. Contrary to feeling supported, the participants reported feeling neglected in their childhood years and perceived a lack of support from their parents. It appears that as they aged the participants’ appraisal of the situation changed, seemingly linked to their development and personality characteristics. This finding has implications for the type of support that would be required by neurotypical siblings across their developmental ages and stages. Participant 7 reflects on this transition when she reports that although she was the youngest in her family of several siblings, most of the attention in the house was afforded to her older brother who was diagnosed with Level 3 ASD. While in her early and mid-childhood years, she perceived the unequal distribution as unfair; as a late adolescent, she understood that her brother required more attention. She reflects as follows:

“It was more a matter of…we’ve got more important issues to look at. Your brother’s condition, everything about him is more important; and that drew her [mother’s] attention more than anything else. So often I would feel like…I could have been on drugs you know, I could have been a rebel, I could have been pregnant by now. You’ve never spoken to me about sex, you’ve never spoken to me about condoms and boys… We learnt to understand, that okay, she’s a single parent, there’s a lot on her head” (Participant 7).

This mirrors findings from previous Euro-based research where neurotypical siblings reported feeling that minimal resources were directed to their needs due to high amounts of time and money spent on their siblings diagnosed with ASD (33). The above results suggest that these South African participants processed their feelings of neglect as children by engaging their cognitive appraisal as adults. As they develop through adolescence, they use their available cognitive resources to make sense of the family dynamics and their parents’ struggles (43, 62). Through emotion-focused coping, participants developed empathy for their parents’ stress. This allowed them to understand why they had limited support available to them and created a space for them to shift their experiences of stress to feelings of compassion and higher sense of emotional control (50).

A few participants (*n* = 3, 37.5%) further indicated that they found support in other parts of their community, apart from their parents, to avoid “piling on more” on their parents (Participant 2). These included friends, “After complaining and seeing that it’s going nowhere I’d complain to my friends” (Participant 5), and teachers, “The schools I went to had like really amazing teachers that also provided us with counseling without us realizing…so I got a lot of support from them” (Participant 2). This is supported by the reports from the previous studies from Singapore and United Kingdom regions, where people outside the family tended to provide supplementary support to siblings of children with ASD (31, 35). These support structures replace the sense of isolation and depression that children could potentially endure at home (44).

#### Parenting their siblings

In needing to care for their sibling diagnosed with ASD, some participants felt they developed prematurely. For example, one of the participants reported that her childhood possibly ended at age eight, when her sibling with a Level 3 ASD diagnosis entered her life. From that age, she realized that her role within her home changed from being a child to becoming a caregiver. A similar finding was reported by other participants who indicated that they understood from a young age that they needed to care for their siblings. As they entered adulthood, they realized that they needed to take over from their parents and become guardians to their sibling diagnosed with ASD.

“As a child I would just fight and swear and tell people to butt off…he’s become like a child to me” (Participant 7).

“Childhood wise…I sacrificed my childhood—like most of the times I couldn’t go out and play with my friends or chill with my friends cause I’d be home babysitting. Because it’s kind of hard to find a nanny that can deal with [sibling’s name] situation because it’s not something that’s common… So most of the time it just me being the head of the house being on that mother role” (Participant 8).

This suggests that some siblings “adopted” the role of parent while children themselves or had parallel roles to their parents in caring for their siblings, resulting in feelings of stress. The feelings of stress are argued by Foster, Hagan, and Brooks–Gunn (63) to stem from adolescents who have peer–peer and not traditional hierarchical relationships with their parents or are required to be developmentally older than their chronological age. Carrying the responsibility of caring for their ASD diagnosed siblings highlights a social dynamic of some South African families. This may be heightened in cases of the neurotypical child being older, hence parental expectations of caring are heightened (9). Benson and Elder (64) disputed that caring for siblings brings about stress, but the young adults rather enjoy psychosocial maturity due to holding more responsibility. This latter argument, however, relates to late adolescents but not to children in their younger years. Furthermore, it may also speak to comparisons of an American sample compared to one in South Africa. However, the findings in this study correlate with Bitsika, Sharpley, and Mailli’s (26) study, implying that when neurotypical children in South Africa, similarly to those in an Australian context, have siblings with ASD, they are likely to perceive having shortened childhoods.

### Social consequences

The participants in the study perceived that having a sibling with ASD had an impact on their social lives. While this impact was predominantly understood as being negative, some positive gains were also identified. Understanding the social impact of having a sibling with ASD draws on Bronfenbrenner’s bio–ecological theory, where the systems are understood as impacting the proximal processes with the individual, but the individual’s own personality characteristics also impact the interactions (39).

Adverse effects on social lives seem to have been most prevalent in participants’ school environments and with peers. The participants’ fears of being ostracized due to having a sibling with ASD was not only owing to their perceptions but also as a result of lived experiences of being bullied and mocked. Participant 7 recalled some incidents at school where she was teased about her “mad brother,” while participant 3 noted that she often got into fights at school after children made painful comments about her brother. This display of aggression can be understood as an attempt at engaging in externalizing behavior as a problem-focused coping strategy (50). The bullying behavior also speaks to the fact that stigma is not only attached to the child diagnosed as having ASD but extends to the sibling as well. This indicates that both the microsystem, as well as beliefs held in the macrosystem affect the neurotypical individuals’ proximal processes. Addressing beliefs in the macrosystem is therefore important if one is to effect change in the microsystem.

According to Erikson, adolescence is a time of identity development, in which good relationships with peers is positively correlated to identity development (65). However, in this study, it was found that the participants tend to become reclusive, as opposed to seeking out social interaction. This behavior was in an effort to deal with the social stress they felt (43, 62), perceiving social interaction as daunting or tiresome. Participant 5 reflected, “I just stopped being social. I didn’t…want to go out anymore. I didn’t want to go meet people… It affected me in the sense that I didn’t make friends,” while Participant 4 notes the following:

“I can’t handle intimate one-on-one like communication. I can’t handle having a female sleepover and sharing the same bed… But clearly, it’s a conscious effort to be like this… I guess people with siblings—normal siblings—they don’t notice like the…intimacy level is much more different than mine” (Participant 4).

Where participants did engage in social interaction, this appeared to be colored with a desire to “hide” the existence of the sibling with ASD, and caution in trusting others. Hiding the existence of their sibling was achieved in one of two ways. The first was to avoid interaction that would require reciprocation. This means that participants chose not to engage in play dates and visits at friends’ homes, as they would later be required to invite their friends to their homes. In cases when neurotypical siblings did invite friends to their homes, they avoided physical proximity to their sibling with the assistance of their parents. These reports coincide with findings from Ferraioli and Harris (34), where siblings’ feelings of embarrassment resulted in less social engagements with their friends in both their home and outside environments. The Petalas et al. (31) and Stampoltzis et al. (32) studies, conducted among European samples, had similar results with participants reporting feelings of shame as result of their siblings’ disorder. Feelings of embarrassment and insecurity were also reported by participants in this study, hindering the disclosure of having a sibling on the spectrum with Participant 3 stating “I used to be embarrassed when uhm, I was in primary school,” while Participant 5 commented the following:

“That was a big insecurity part of my life that I have an autistic brother at home… I remember just feeling like he’s sort of like ruining my reputation because now I was just known as the girl with the little brother whose disabled, and I just felt like nobody will see me for me” (Participant 5).

In these examples, it is the individual’s own personality characteristics that affected the disclosure, and not the microsystem *per se*. Reports from participants indicated that as children, they felt that opening up about their siblings’ ASD would result in them being alienated or viewed as odd. This is illustrated in the below quotes.

While the participants were hesitant in sharing about their sibling with ASD, in cases where they shared with peers about their sibling’s ASD diagnosis, it was an indication of the strength of their friendships. They considered the people who accepted their brother or sister as genuine friends. Some also alluded to the idea that sharing about the ASD diagnosed sibling could have strengthened their friendships with Participants 4 and 5, respectively, sharing, “I don’t talk about it…but my one close girlfriend…she’s like my sister and so we talk about it often” (Participant 4), “I think I probably would have had a lot of…non-true friendships…Because having him really tested a lot of friendships” (Participant 5). Sharing and feeling safe in talking about their siblings’ ASD diagnosis was also assessed as beneficial (45) because it became a marker of the strength of friendships.

### Acceptance

Analysis of the findings indicated acceptance as a vital and recurrent theme (*n* = 7, 87.5%). Preceding research has reported similar findings, where siblings’ adjustment was noted to correlate with acceptance of their siblings on the spectrum (30). These studies argued that when neurotypical children were willing to accept the responsibility and stress of their sibling diagnosed with ASD, positive acceptance afforded them more adjustment potential and higher self-esteem (30, 32). In this study, the participants reported coming to acceptance through two mechanisms, these were through normalization and applying their faith. It is noteworthy that the participants reflected on acceptance as emerging adults, having developed the ability to evaluate their experiences in a more holistic way (66, 67).

#### Normalization

The previous themes illustrate how the participants experienced different stressors as a consequence of having a brother or sister on the spectrum. Having said that, many added that they were not able to perceive their lives without their brother or sister who was diagnosed with ASD. While they occasionally became frustrated at seeing the consequences of what an ASD diagnosis meant for their siblings, themselves and their family, many could not picture their sibling without autism. These experiences along with the responsibilities that came with them were something they had become accustomed to with comments such as, “I can’t picture any other life” (Participant 4) and “When you’re used to it, it’s the norm. It’s who he’s always been so it’s not a problem… it is what it is” (Participant 1).

The participants had only known life with a sibling on the spectrum. Although they experienced hardships linked to their siblings’ ASD diagnosis, the consistency of these hardships seems to have led to them secondarily appraising that they had the resources available to cope with the related stress (43). This is a unique finding that is not found in other research. The participants of this study indicated an internal locus of control and use of emotion-focused coping, suggesting an attribute of the neurotypical individuals in this study. This further indicates that within South Africa, i.e., the particular culture of these participants, coping through family struggles by accepting their siblings’ ASD was a parental expectation, and a means of avoiding conflicts between the family members.

#### The role of faith

Several interviewees (*n* = 5, 62.5%) mentioned that they relied on their faith as a means of dealing with the ASD and its consequences. Few of them (*n* = 3, 37.5%) explained that not only did religion assist them through the difficulties but also saw themselves or their family as purposefully chosen to have a child with ASD as part of their system. These belief systems resulted in them understanding their stressors as a root of growth and cohesion for them and/or their family. This is contrary to the beliefs related to the etiology of disability in African countries, as reported by Mutasa and Ruhode (68) and Reynolds (69). In their studies, the authors report that disability is understood as being related to spiritual elements such as witchcraft, referred to as “non-biological based belief” (70. p.153). Instead, the participants reported, “I cope by just having faith, and you know believing in the family; and that if someone has to go through it at least it’s us, we have empathy for it” (Participant 4). Similarly, the below was stated:

“I do believe that God gives us challenges that he knows we can face. And maybe you know he realized that especially our family; maybe he saw that you know what there’s a lot of ignorance going on here” (Participant 2).

By making sense of their challenges using faith, the results highlight what Folkman and Lazarus (49) argued, that positive emotion-focused coping can afford people the feeling of being in control of their struggles. When faith was a coping strategy in some of the families, their strategies to cope living with a child diagnosed with ASD buffered the stresses they experienced (34).

### Identity development

The last theme that emerged from participants was identity development. The sub-themes for this theme were ASD as core to their identity development, and siblings’ influence on chosen life paths.

#### Autism spectrum disorder as core to identity development

The participants felt that their siblings having autism became one of the central factors to their identity development. One participant stated that her brother having been diagnosed with ASD, translated to her equally having autism with him. This emulates other participants who indicated that having a sibling with ASD became a core part of their identity. This was not only an identity factor for individuals but also became a trait characteristic to their families, where they carried autism with them.

“I am the sibling of a brother with autism. And even though my brother isn’t here, I’m still the sibling of a brother with autism” (Participant 7).

“So now it’s like a fact that comes along with me” (Participant 5).

The finding appears to be distinctive to this study and not reported in previous research. This may indicate that the collectivist inclined societal values of South African participants results in their siblings with ASD significantly contributing to their personal identity. They reported having developed positive character traits including patience, tolerance, or acceptance of difference. Thus, indicating that proximal processes impacted the development of personality characteristics.

“In a social sense, it’s made me more aware of different people and a bit more accepting. You know to having one set of social expectations and knowing that [not] everybody’s got to conform” (Participant 1).

“It impacted me deeply I guess uhm it really shaped who I was… maybe I would be a bit more selfish…maybe I’d be a bit less kind to people you know if it wasn’t for him” (Participant 4).

The above is consistent with previous research where other demographics of neurotypical children felt their stress and general experiences related to their siblings’ ASD diagnosis, resulted in them developing personal traits of compassion, tolerance, maturity, patience, increased responsibility, and love for social justice. It also contributed positively to their self-concept (32, 70), highlighting that the personality characteristics of children in Western and a South African context are influenced by their atypical siblings.

#### Influence on chosen life path

The participants also indicated that their experiences with their siblings influenced their desire to help others. In numerous cases (*n* = 5, 62.5%), their sibling contributed to their career choices. Participant 1 reported that her choice of study was informed by her having developed an increased awareness of disabilities and disorders that children and people live with. Similar life choices are explained by other participants below.

“It is kind of like giving up a life, but I mean it’s the right thing to do I guess… It’s just something that just needs to be fulfilled you know in life we’ll always have…our destinies… I’m just like ya God you just gave me this purpose and there is a way for it, there is a market, there is a need. I can flourish in it. So I am good at it, and if I have so much knowledge about it I just can’t let it go to waste. Ya, so that’s why and it’s okay” (Participant 4).

“Everything—the person I’ve become, the…character I’ve become, the life path I’m choosing, the school I’d like to own, the difference I’d like to make—it’s all my brother” (Participant 7).

Findings of siblings on the spectrum being key to the identity development of their neurotypical siblings supports what Chan and Goh (35) illustrated. In their study the authors commented that although participants shared their painful and stressful experiences of loneliness, lessened support, etc., many did not view themselves as victims of their siblings’ ASD. Instead, they made positive meaning of these experiences. Following the Cognitive Mediational Model, this indicates that coping, more often emotion-focused coping, allows neurotypical children with siblings diagnosed with ASD, to take control of their lives regardless of their stress-laden experiences. Noting that Chan and Goh’s study was conducted on a Singaporean sample, i.e., a predominantly collectivist nation, this developmental trajectory may be attributed to neurotypical siblings from collectivist cultures (70).

Some of the above findings mirror the previous research conducted, however, others were novel, suggesting nuances particular to the South African demographic. This has implications for praxis in support of neurotypical siblings of ASD diagnosed individuals and their families.

### Strengths and limitations

One of the strengths of the study was that the participants were individuals within the late adolescent stage, where increased perspective-taking and reflection were possible. They were thus able to reflect on their experiences while identifying changes in their perceptions across time. However, with a focus on late adolescents the study did not capture the experiences of younger children. Given the variation in cognitive capacities, it is recommended that further studies be conducted on neurotypical children of age group 5–18, where the development is characterized by more egocentrism, and children may be navigating more disruptive stages of the ASD diagnosis previously reported by neurotypical children (71) Additionally, the participants of this study were all females, hence the data from diverse gender South African samples will be beneficial. This is to be noted that while this refers to a different demographic, Euro–American sisters to children with ASD reported fewer negative experiences than brothers (72, 73).

## Conclusion

This article has sought to gain an understanding of the experiences of neurotypical siblings being raised alongside a sibling diagnosed with ASD within the typically collectivist South African context employing an interpretive phenomenological analysis research design This study demonstrated the psychological, emotional, and social impact of being raised alongside a sibling diagnosed with ASD. It is evident that the impact was felt across all dimensions of the bio–ecological theory, i.e., proximal processes, personality characteristics, context, and time. Important to note is the changes in personality characteristics as a result of the experience as well as the self-imposed restriction on interactions within the microsystem. Neurotypical individuals developed positive personality characteristics from lived experiences related to their ASD diagnosed siblings. Having said that, numerous stress-laden experiences made evident the need to provide support to the neurotypical siblings and their surrounding systems. Suggested support is through increased knowledge around ASD with the aim of minimizing the challenges caused by stigmatization of siblings or diverse community members, as this affects perceptions and socialization with people outside of the families affected by ASD (8). This could be achieved through supportive programs and awareness campaigns that encourage sharing experiences and creating opportunities of normalization of the disorder in diverse contexts such as schools, faith institutions, and clinics. Additionally, the finding that individuals receive support from individuals outside of their nuclear families is an important one in the collectivist society of South Africa where personality development can be significantly influenced by people beyond the nuclear family (19). Thus, support groups for children and persons in their microsystem who are affected by ASD may offer spaces to develop emotional coping.

## Data availability statement

The raw data supporting the conclusions of this article will be made available by the authors, without undue reservation.

## Ethics statement

The studies involving human participants were reviewed and approved by University of the Witwatersrand Human Research Ethics Committee (Non-medical). The patients/participants provided their written informed consent to participate in this study.

## Author contributions

Both authors listed have made a substantial, direct, and intellectual contribution to the work, and approved it for publication.

## Conflict of interest

The authors declare that the research was conducted in the absence of any commercial or financial relationships that could be construed as a potential conflict of interest.

## Publisher’s note

All claims expressed in this article are solely those of the authors and do not necessarily represent those of their affiliated organizations, or those of the publisher, the editors and the reviewers. Any product that may be evaluated in this article, or claim that may be made by its manufacturer, is not guaranteed or endorsed by the publisher.

## Appendix

### Appendix A | Interview schedule

**Demographic/Background questions**

1. Can you tell me your current age?
2. What ethnicity are you?
3. With whom do you live?
4. Did your parents work when you growing up? Who worked in the house? Where did the household income come from?
5. Can you tell me briefly about your relationship with people in your household now and when you were younger?
6. I would just like to confirm that your brother/sister is diagnosed with autism? How old is he/she?
7. What gender is he/she?
8. When did you begin to know that he/she is diagnosed with autism?

**Relationship with their sibling**

1. Can you explain what your relationship with your brother/sister is like?
2. What activities do you and did you do together?
3. How do you feel about your brother/sister having autism?
4. Through your life, how have you generally managed with your brother/sister having autism?

**Autism spectrum disorder in the family**

1. Growing up, what responsibilities did you both have in the house?
2. How do you think your life has been affected by your brother’s/sister’s autism?
3. Did/does your brother/sister attend therapy for their autism? Did/have you ever attended that therapy too? *(If relevant)* Can you tell me about how you felt about that therapy?
4. What has your relationship with your parents been like? How was it like in the past and how it is it like now?
5. How was the support you got in the house for your personal things (school, boys/girls, social life, other important things)?

**Autism spectrum disorder in relation to their social selves**

1. How has your brother’s/sister’s autism affected your friendships?
2. How did your interaction with your brother/sister influence your socialization in primary school, high school, and now?

**A possible life without ASD**

1. Does having a sibling with autism make your life different than it would have been if he/she did not have autism? If so, in what way? How would you have spent time with him/her?
2. Do you think that if your sibling did not have autism, it would have affected your relationship with your parents differently? If so, how?

**Debrief**

1. How are you feeling after having shared your experiences today?
2. Has this process brought about any feelings of discomfort that you would like to further talk about?
3. Is there something more you would like to share?
